# C14ORF166 overexpression is associated with pelvic lymph node metastasis and poor prognosis in uterine cervical cancer

**DOI:** 10.1007/s13277-015-3806-3

**Published:** 2015-07-29

**Authors:** Weijing Zhang, Jianping Ou, Fangyong Lei, Teng Hou, Shu Wu, Chunhao Niu, Liqun Xu, Yanna Zhang

**Affiliations:** 1 Department of Gynecologic Oncology; State Key Laboratory of Oncology in South China; Collaborative Innovation Center for Cancer Medicine, Cancer Center, Sun Yat-Sen University, No. 651, Dongfeng Road East, Guangzhou, 510060 People’s Republic of China; 2 Center for Reproductive Medicine, The Third Affiliated Hospital of Sun Yat-Sen University, No. 600, Tian He Road, GuangZhou, 510630 People’s Republic of China; 3 State Key Laboratory of Oncology in South China, Collaborative Innovation Center for Cancer Medicine, Guangzhou, 510060 People’s Republic of China; 4 Department of Urology, Wuhan Union Hospital of Huazhong University of Science and Technology, No. 1277, Han Kou Jie Fang Road, Wuhan, 430022 People’s Republic of China; 5 Department of Gynecology, Women and Children Hospital of Guangdong Province, No. 13, Guang Yuan Road, Guangzhou, 510060 People’s Republic of China

**Keywords:** C14ORF166, Cervical cancer, Lymph node metastasis, Prognosis, Biomarker

## Abstract

C14ORF166 (chromosome 14 open reading frame 166) is a transcriptional repressor related to the regulation of centrosome architecture. However, the role of C14ORF166 in the development and progression of cancer remains largely unknown. The aim of this study was to investigate the expression and clinicopathological significance of C14ORF166 in cervical cancer. C14ORF166 expression was analyzed using quantitative real-time PCR (RT-PCR) and Western blotting in cervical cancer cell lines and eight paired cervical cancer samples and the adjacent normal tissues. Immunohistochemistry was used to analyze C14ORF166 protein expression in 148 clinicopathologically characterized cervical cancer specimens. Statistical analyses were performed to evaluate the relationship between the expression of C14ORF166 and clinicopathologic features and prognosis. C14ORF166 mRNA and protein expression were significantly upregulated in cervical cancer cell lines and tissue samples (*P* < 0.05). Immunohistochemical analysis revealed a high expression of C14ORF166 was observed in 39.9 % (59/148) of the cervical cancer specimens; the remaining samples expressed low levels or did not express any detectable C14ORF166. The chi-square test indicated that high-level expression of C14ORF166 was significantly associated with International Federation of Gynecology and Obstetrics (FIGO) stage (*P* < 0.001), vital status (*P* = 0.026), tumor size (*P* = 0.034), serum squamous cell carcinoma antigen level (SCC-Ag; *P* = 0.035), and pelvic lymph node metastasis (*P* < 0.001). Patients with highly expressed C14ORF166 showed a tendency to receive postoperative chemotherapy (*P* = 0.005) and postoperative radiation (*P* = 0.008). Furthermore, high C14ORF166 expression was associated with poorer overall survival compared to low C14ORF166 expression, and C14ORF166 was a significant prognostic factor in univariate and multivariate analysis (*P* < 0.05). High C14ORF166 expression had prognostic value for poor outcome in cervical cancer. C14ORF166 may represent a biomarker of pelvic lymph node metastasis and enable the identification of high-risk patients along with selection of appropriate treatment strategies.

## Introduction

Cervical cancer is the third most common malignancy among females worldwide, with an estimated global incidence of more than 500,000 new cases and 260,000 deaths every year [1]. Cervical cancer is prevalent in developing countries, where it remains a major cause of mortality among females [2]. Viral infection, genetic susceptibility, and environmental factors are associated with the etiology of cervical cancer. The development of cervical lesions from cervical intraepithelial neoplasia (CIN) to cervical cancer is a complicated process that is initiated by persistent infection with high-risk types of human papillomavirus (HPV) [3]. Altered expression of a variety of oncogenes and tumor suppressor genes has also been associated with cervical cancer [4]. However, the precise mechanisms that regulate the pathogenesis of cervical cancer are poorly characterized, and a specific genetic signature to predict prognosis has not yet been identified in cervical cancer. Conventional clinical, diagnostic, and pathological variables such as International Federation of Gynecology and Obstetrics (FIGO) stage, tumor size, and the depth of infiltration are still used extensively to predict prognosis but are not sufficiently reliable to predict clinical outcome [5]. Patients with lymph node metastasis have a higher rate of mortality than patients without lymph node metastasis [6], and assessment of lymph node metastasis provides important information for determining the appropriate treatment approach [7]. Hence, it would be of clinical significance to identify biomarkers of lymph node metastasis or prognostic factors in patients with cervical cancer.

*C14ORF166* is located on chromosome 14 at 14q22.1 and encodes a highly conserved 28-kDa protein. *C14ORF166* was originally identified as an influenza A virus-associated gene and may modulate transcription by interacting with various transactivators [8]. C14ORF166 has been suggested to play a prominent role in the fate of nuclear and cytoplasmic RNA. The C14ORF166-DDX1-HSPC117-FAM98B complex is present in both cellular compartments and transports RNA molecules between the nucleus and the cytoplasm [9]. Interestingly, overexpression of C14ORF166 has been observed in a variety of human cancers. Wang et al. [10] reported that C14ORF166 is highly expressed in a variety of brain tumors and is differentially expressed during normal brain development. Recently, C14ORF166 was reported to be a potential biomarker for metastasis in patients with human pancreatic carcinoma [11, 12]. Along with acylglycerol kinase (AGK), C14ORF166 has been implicated in the JAK2/STAT3 signaling pathway in esophageal squamous cells [13]. The JAK2/STAT3 pathway has been reported to play a crucial role in regulating the progression of cervical cancer [14, 15]. However, the protein expression level and role of C14ORF166 in cervical cancer have not been investigated.

In the current study, we aimed to explore the expression of C14ORF166 in cervical cell lines and human cervical tissues. Moreover, we investigated the association between the expression of C14ORF166 protein and clinical significances and survival outcomes of a cohort of 148 patients with cervical cancer.

## Methods

### Cell lines

Patient consent was obtained prior to the use of the clinical materials for research purposes, and the patient consent and protocol were approved by Sun Yat-Sen University Cancer Center Institutional Review Board. A primary culture of normal cervical epithelial cells was established from a biopsy of noncancerous cervical epithelium and was cultured in complete Keratinocyte-SFM medium (Invitrogen, Carlsbad, CA, USA). The cervical cancer cell lines HeLa, SiHa, C33A, MS751, CasKi, HeLa229, and HCC94 were cultured in RPMI-1640 medium (GIBCO BRL), and ME-180 was cultured in DMEM medium (Gibco, Grand Island, NY, USA), all supplemented with 10 % fetal bovine serum (HyClone Laboratories, Logan, UT).

### Patients and tissue samples

For real-time PCR (RT-PCR) and Western blot analysis, we collected 8 paired cervical cancers and adjacent normal tissues from the patients who underwent surgery between 2013 and 2014. A total of 148 cases of paraffin-embedded cervical cancer samples had been clinically and histologically diagnosed at the Sun Yat-Sen University Cancer Center (Guangzhou, China) from 2001 to 2005. Clinical and clinicopathological classification and staging of all the patients were defined according to the International Federation of Gynecology and Obstetrics (FIGO) criteria (Pecorelli, 2009): 51 were respectively allocated to stage I; 80 to stage II; 15 to stage III; and 2 to stage IV. The patients’ age ranged from 25 to 68 years (mean, 42.57 years). Clinicopathological characteristics of the tumor cases are presented in Table 1. The follow-up time of the cohort ranged from 2 to 132 months, with a median follow-up time of 51.65 months.

**Table 1:** Clinicopathological characteristics and tumor expression of *C14ORF166* in patients with cervical cancer

Characteristic	Number of cases (%)
Age (years)	
<50	114 (77.0)
≥50	34 (23.0)
FIGO stage	
I	51 (34.5)
II	80 (54.0)
III	15 (10.1)
IV	2 (1.4)
Histological type	
Squamous carcinoma	143 (96.6)
Adenocarcinoma	5 (3.4)
Pelvic lymph node metastasis	
No	115 (77.7)
Yes	33 (22.3)
Expression of C14ORF166	
Low or none	89 (60.1)
High	59 (39.9)
Tumor size, cm	
<4 cm	71 (48.0)
≥4 cm	77 (52.0)
Tumor recurrence	
No	133 (89.9)
Yes	15 (10.1)
Vital status (at last follow-up)	
Alive	127 (85.8)
Dead	21 (14.2)
Differentiation grade	
G1	3 (2.0)
G2	59 (39.9)
G3	86 (58.1)
Postoperative chemotherapy	
No	47 (31.8)
Yes	101 (68.2)
Postoperative radiation	
No	92 (62.2)
Yes	56 (37.8)
Squamous cell carcinoma antigen, ng/ml	
≤1.5	93 (62.8)
>1.5	55 (37.2)

### Real-time PCR

Total RNA samples from cell lines and fresh surgical cervical cancer tissue were isolated using Trizol reagent (Invitrogen, Carlsbad, CA, USA) according to the manufacturer’s instructions. The extracted RNA was pretreated with RNase-free DNase, and approximately 2 μg of RNA from each sample was used for cDNA synthesis primed with random hexamers. For the PCR amplification of *C14ORF166* cDNA, an initial amplification step using C14ORF166-specific primers was performed with denaturation at 95 °C for 10 min. This was followed by 28 denaturation cycles at 95 °C for 60 s, primer annealing at 58 °C for 30 s, and a primer extension phase at 72 °C for 30 s. Upon the completion of the cycling steps, a final extension step at 72 °C for 5 min was performed before the reaction mixture was stored at 4 °C. Real-time PCR was then employed to determine the increase of *C14ORF166* mRNA in each of the primary cervical tumors relative to the paired normal cervical tissue taken from the same patient. The primers were designed using Primer Express v 2.0 software (Applied Biosystems). The *C14ORF166* sense primer was 5′-TGCATTGTCAGCAGTTTTTGA-3′, and the antisense primer was 5′-TGACTGGCTTCTTGGTTTAGC-3′. For the *GADPH* gene, the sense primer was 5′-TTGAGGTCAATGAAGGGGTC-3′, and the antisense primer was 5′-GAAGGTGAAGGTCGGAGTCA-3′. Expression data were normalized to the geometric mean of the housekeeping gene GADPH to control the variability in expression levels and calculated as 2-[(Ct of C14ORF166)—(Ct of GADPH)], where Ct represents the threshold cycle for each transcript.

### Western blotting

Sample preparation for immunoblotting was performed as previously described. Cells at 80 to 90 % confluence were washed twice with ice-cold phosphate-buffered saline (PBS) and lysed on ice in radio immunoprecipitation assay buffer (RIPA; Cell Signaling Technology, Danvers, MA) containing complete protease inhibitor cocktail (Roche Applied Sciences, Mannheim, Germany). Fresh tissue samples were ground to powder in liquid nitrogen and lysed with SDS-PAGE sample buffer. Protein concentration was determined by the Bradford assay (Bio-Rad Laboratories, Hercules, CA). Equal amounts of proteins (30 μg) were separated electrophoretically on 10.5 % SDS/polyacrylamide gels and transferred onto PVDF membranes (Immobilon P, Millipore, Bedford, MA). Membranes were blocked with 5 % fat-free milk in Tris-buffered saline containing 0.1 % Tween-20 (TBS-T) for 1 h at room temperature. Membranes were probed with an anti-C14ORF166 rabbit polyclonal antibody (1:2000, Proteintech) overnight at 4 °C. After washing with TBS-T, the membrane was incubated with a secondary antibody against mouse immunoglobulin G. The membrane was washed, and protein was detected by enhanced chemiluminescence (Amersham Pharmacia Biotech) according to the manufacturer’s instructions. An anti-α-tubulin mouse monoclonal antibody (1:1000; Santa Cruz Biotechnology, Santa Cruz, CA) was used to confirm equal loading.

### Immunohistochemical analysis

Immunohistochemistry was done to examine C14ORF166 expression in 148 human cervical cancer specimens. Briefly, paraffin-embedded tissues were cut into 4-Am sections and baked at 60 °C for 1 h. The sections were deparaffinized with xylenes, rehydrated, then submerged into citrate antigenic retrieval buffer and microwaved for antigenic retrieval. The samples were then treated with 3 % hydrogen peroxide in methanol to quench the endogenous peroxidase activity, followed by incubation with 1 % bovine serum albumin to block the nonspecific binding. Finally, the sections were incubated with anti-C14ORF166 rabbit polyclonal antibody (1:200, Proteintech) overnight at 4 °C. For negative controls, the primary antibody was replaced by normal goat serum. After washing, the tissue sections were then incubated with the biotinylated antimouse secondary antibody (Abcam), followed by further incubation with streptavidin-horseradish-peroxidase complex (Abcam). The tissue sections were immersed in 3-amino-9-ethyl carbazole, counterstained with 10 % Mayer’s hematoxylin, dehydrated, and mounted in crystal mount.

The degree of immunostaining of formalin-fixed, paraffin-embedded sections was reviewed and scored by three independent observers who were blinded to the histopathological features and patient data of the samples. The scores given by the three independent investigators were averaged and based on both the proportion of positively stained tumor cells and the intensity of staining. The intensity of protein expression was recorded as follows: 0 (no staining), 1 (weak staining, light yellow), 2 (moderate staining, yellowish brown), and 3 (strong staining, brown). The proportion of tumor cells was scored as follows: 1 (<10 % positive tumor cells), 2 (10–50 % positive tumor cells), 3 (50–75 % positive tumor cells), and 4 (>75 % positive tumor cells). The staining intensity and the proportion of positive cell scores for each section were multiplied (scored as 0, 1, 2, 3, 4, 6, 8, 9, or 12). Using this method of assessment, we evaluated the expression of C14ORF166 in cervical cancer tissue. Cut-off values for C14ORF166 were chosen on the basis of a measure of heterogeneity using the log-rank test with respect to overall survival (OS). An optimal cut-off value was identified as follows: a staining index score of >6 was utilized to define tumors with high C14ORF166 expression and ≤6 suggested low C14ORF166 expression. To account for the inconsistencies in IHC stain intensities, the mean optical density (MOD) method, which was used for the scoring of the staining intensity, was applied in the current study.

### Statistical analysis

All statistical analyses were conducted using the SPSS 16.0 statistical software packages. In the real-time PCR and Western blot analysis, the significance of mRNA and protein expression between cervical cancers and the adjacent normal tissues was analyzed by *t* test. We analyzed the relationship between expression of C14ORF166 protein, clinicopathologic features, and the clinical prognosis. The chi-square test and Fisher’s exact test were used to analyze the relationship between C14ORF166 expression and clinicopathological characteristics. Bivariate correlations between study variables were calculated by association analysis. Survival curves were plotted by the Kaplan–Meier method and compared using the log-rank test. Using the Cox proportional hazards regression model in the univariate and multivariate analysis, the significance of various variables which were mentioned above for survival was analyzed to predict prognosis in clinical practice. In all cases, a *P* value of less than 0.05 was considered to be statistically significant.

## Results

### C14ORF166 is overexpressed in cervical cancer cell lines

**Fig. 1:**
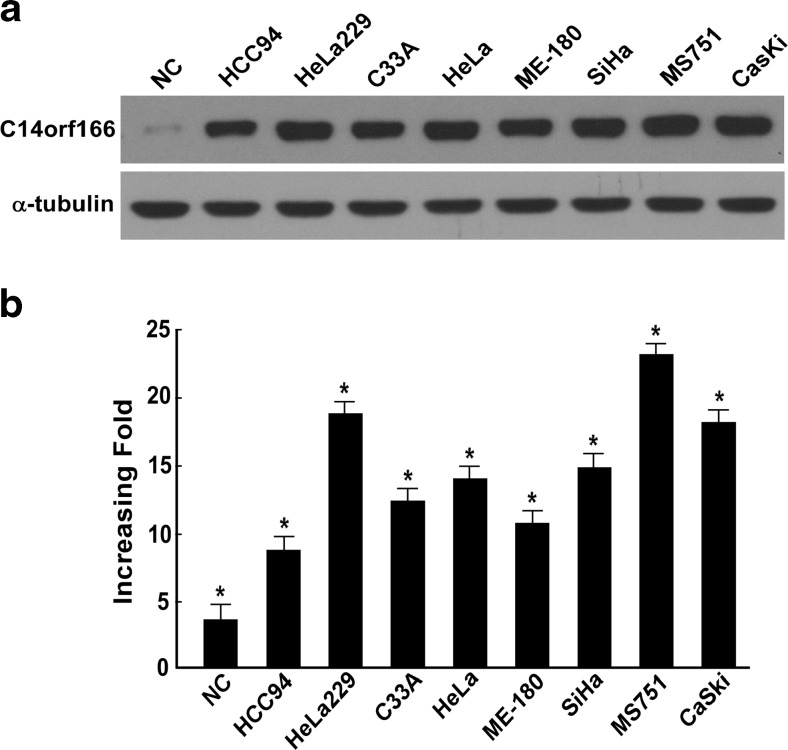
Overexpression of *C14ORF166* mRNA and protein in cervical cancer cell lines. **a**, **b** Expression of *C14ORF166* mRNA and protein in cervical cancer cell lines (HCC94, HeLa229, C33A, HeLa, ME-180, SiHa, MS751, CasKi) and normal cervical cell lines were examined by Western blotting (**a**) and qPCR (**b**). Expression levels were normalized against GAPDH respectively. *Error bars* represent the standard deviation of the mean (SD) calculated from three parallel experiments. *p < 0.05

RT-PCR and Western blotting were performed to determine C14ORF166 mRNA and protein expression in eight cervical cancer cell lines (HeLa, SiHa, C33A, MS751, CasKi, HeLa229, HCC94, and ME-180) and normal cervical cell lines (NC). As shown in Fig. 1a and b, both C14ORF166 mRNA and protein were expressed at high levels in all cervical cancer cell lines tested compared to normal cervical lines.

### C14ORF166 is overexpressed in human cervical cancer

**Fig. 2:**
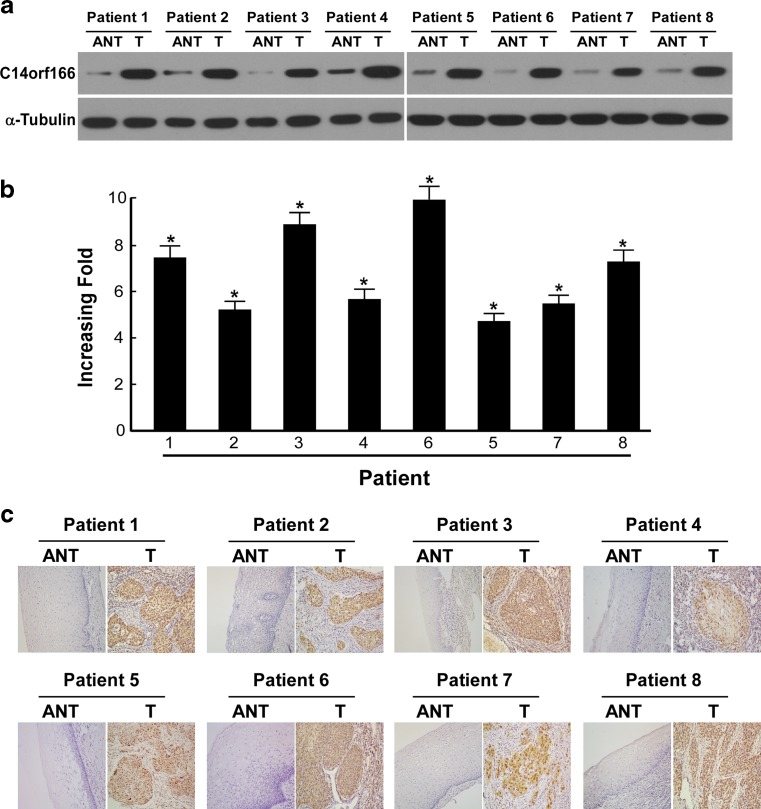
Overexpression of *C14ORF166* mRNA and protein in cervical cancer tissues. **a** Representative images of Western blotting analyses of *C14ORF166* protein expression in eight matched pairs of cervical cancer (T) and adjacent noncancerous tissues. The expression level was normalized by α-tubulin expression. **b** Average T/N ratios of *C14ORF166* mRNA expression in paired cervical cancer (T) and adjacent noncancerous tissues (N) were quantified by qPCR and normalized against GAPDH. *Error bars* represent the standard deviation of the mean (SD) calculated from three parallel experiments. **c** Immunohistochemical assay of *C14ORF166* protein expression in eight pairs of matched cervical cancer tissues. *p < 0.05

To evaluate the expression of C14ORF166 in human cervical cancer, RT-PCR and Western blotting were performed on cervical cancer specimens of eight patients and their paired adjacent noncancerous tissues. All eight human cervical cancer tissue samples expressed high levels of C14ORF166 mRNA and protein; whereas, C14ORF166 was expressed at low levels in the adjacent normal tissues (Fig. 2a, b).

**Fig. 3:**
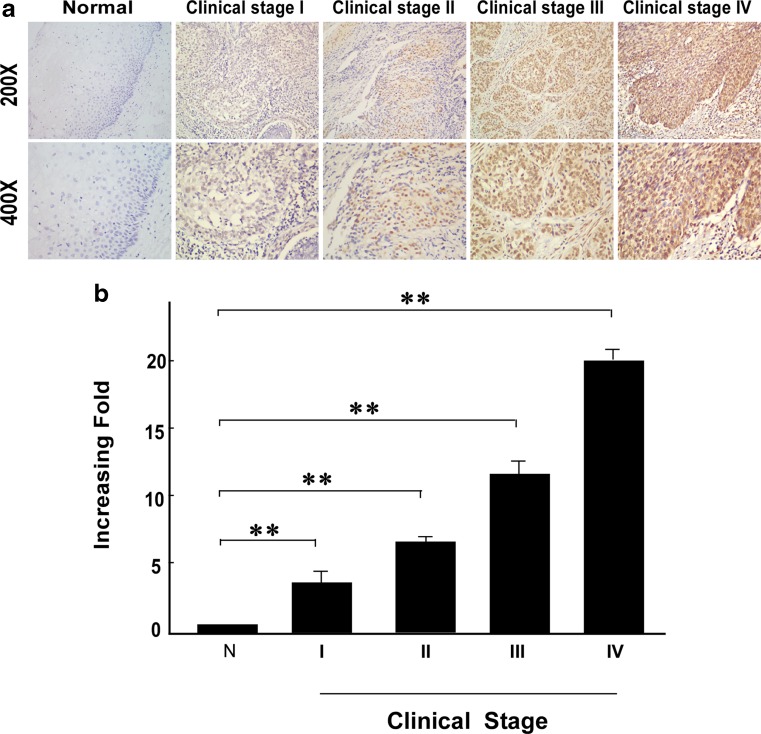
**a** The expression of *C14ORF166* protein in cervical cancer tissues from patients at different clinical stages. **b** The average MOD of *C14ORF166* staining between the normal cervical tissues (four cases) and different clinical stage cervical tissues (randomly picked 10 cases per stage) were statistically quantified. The average MOD of *C14ORF166* staining increases as cervical cancer progresses to more advanced stages (*P* < 0.001). *Error bars* represent mean ± SD from three independent experiments.

C14ORF166 protein expression was assessed in 148 paraffin-embedded archived cervical cancer tissues by immunohistochemical staining using an antibody against human C14ORF166. Either no immunoreactivity or only weak staining was observed in the adjacent noncancerous tissues and normal cervical tissues; whereas, C14ORF166 was expressed at high levels in the cervical cancer tissues (Fig. 2c). In the tumor tissues, C14ORF166 was primarily localized in the tumor cell nuclei with strong cytoplasmic staining occasionally observed (Fig. 3a). High levels of C14ORF166 protein expression were observed in 59/148 patients (39.9 %), and weak or no staining was observed in 89 patients (60.1 %; Table 1). Furthermore, the statistical analyses of the average mean optical density (MOD) of C14ORF166 staining in normal cervical tissues and cervical cancer specimens at different clinical stages revealed that C14ORF166 expression increased with advancing FIGO stage in cervical cancer (Fig. 3).

### C14ORF166 expression correlates with the clinicopathologic features of cervical cancer

The correlation between expression of C14ORF166 protein and the clinicopathological features of cervical cancer were analyzed using the chi-square test and Fisher’s exact test (Table 2). High expression of C14ORF166 protein was significantly associated with FIGO stage (*P* < 0.001), vital status (*P* = 0.026), tumor size (*P* = 0.034), pelvic lymph node metastasis (*P* < 0.001), and serum squamous cell carcinoma antigen level (*P* = 0.035). Patients with highly expressed C14ORF166 showed a tendency to receive postoperative chemotherapy (*P* = 0.005) and postoperative radiation (*P* = 0.008). However, there were no statistically significant correlations between C14ORF166 protein expression and other clinicopathologic features such as patient age, histological differentiation, and recurrence.

**Table 2:** Correlation between *C14ORF166* expression and the clinicopathologic features of cervical cancer

Characteristic		Total	C14ORF166		Chi-squared test *P* value	Fisher’s exact test *P* value
			No or weak expression	Moderate or strong expression		
Age (years)	<50	114	67 (45.3)	47 (31.8)	0.535	0.557
	≥50	34	22 (14.9)	12 (8.1)		
FIGO stage	I	51	46 (31.1)	5 (3.4)	<0.001	–
	II	80	42 (28.4)	38 (25.7)		
	III	15	1 (0.7)	14 (9.4)		
	IV	2	0 (0)	2 (1.4)		
Pelvic lymph node metastasis	Absent	115	85 (57.4)	30 (20.3)	<0.001	<0.001
	Present	33	4 (2.7)	29 (19.6)		
Tumor size	<4 cm	71	49 (33.1)	22 (14.9)	0.034	0.044
	≥4 cm	77	40 (27.0)	37 (25.0)		
Tumor recurrence	No	133	81 (54.0)	52 (36.5)	0.570	0.588
	Yes	15	8 (5.4)	7 (4.1)		
Vital status (at last follow-up)	Alive	127	81 (54.7)	46 (31.1)	0.026	0.032
	Dead	21	8 (5.4)	13 (8.8)		
Differentiation	G1	3	2 (1.4)	1 (0.7)	0.952	–
	G2	59	36 (24.3)	23 (15.5)		
	G3	86	51 (34.5)	35 (23.6)		
Postoperative chemotherapy	No	47	36 (24.3)	11 (7.4)	0.005	0.007
	Yes	101	53 (35.8)	48 (32.4)		
Postoperative radiation	No	92	63 (42.6)	29 (19.6)	0.008	0.010
	Yes	56	26 (17.5)	30 (20.3)		
Squamous cell carcinoma antigen, ng/ml	≤1.5	93	62 (41.9)	31 (21.0)	0.035	0.039
	>1.5	55	27 (18.2)	28 (18.9)		

Association analysis was performed to confirm the correlations between C14ORF166 expression and the clinicopathological features of cervical cancer. As shown in Table 3, statistically significant correlations were observed between C14ORF166 expression and FIGO stage (0.561; *P* < 0.001), pelvic lymph node metastasis (0.465; *P* < 0.001), tumor size (0.172; *P* = 0.034), and the serum squamous cell carcinoma antigen level (0.171; *P* = 0.035). In addition, patients with highly expressed C14ORF166 showed a tendency to receive postoperative chemotherapy (0.224; *P* = 0.005) and postoperative radiation (0.213; *P* = 0.008).

**Table 3:** Correlation between C14ORF166 expression and the clinicopathological characteristics of patients with cervical cancer

Variable	C14ORF166 expression	
	Association coefficient	*P* value
Age	0.051	0.535
FIGO Stage	0.561	<0.001
Pelvic lymph node metastasis	0.465	<0.001
Tumor size	0.172	0.034
Recurrence	0.047	0.570
Vital status	0.180	0.026
Differentiation grade	0.026	0.952
Postoperative chemotherapy	0.224	0.005
Postoperative radiation	0.213	0.008
Squamous cell carcinoma antigen, ng/ml	0.171	0.035

### Increased C14ORF166 expression is associated with poor prognosis in cervical cancer

**Fig. 4:**
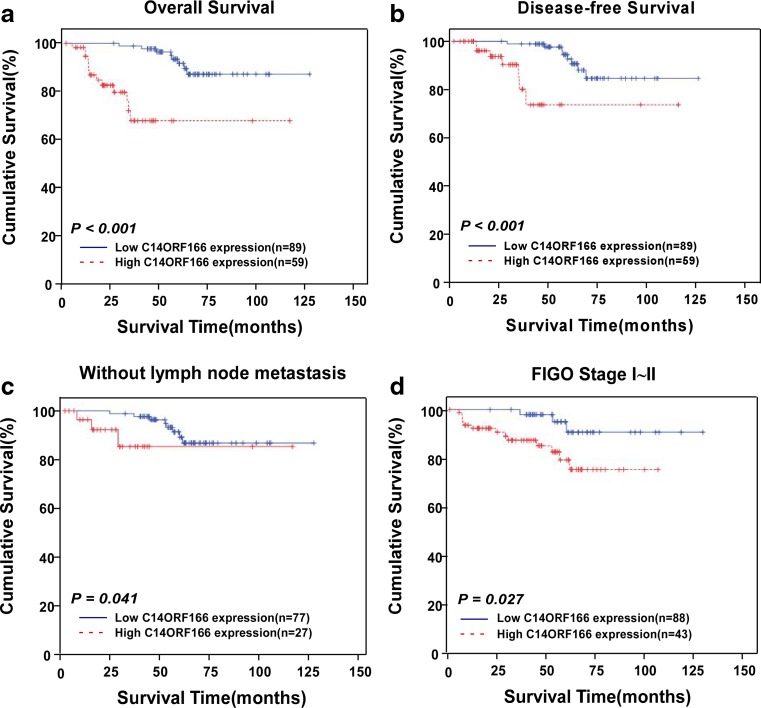
Survival curves of patients with cervical carcinoma, subdivided according to *C14ORF166* protein expression (log-rank test). **a**, **b** OS (**a**) and 5-year DFS (**b**) rates for cases with high *C14ORF166* expression versus those for cases with low *C14ORF166* expression levels in all patients. **c**, **d** OS rate for without lymph node metastasis cases (**c**) and clinical stage I/II cases (**d**) with high *C14ORF166* expression versus those for cases with low *C14ORF166* expression levels.

Kaplan–Meier survival analysis and the log-rank test indicated that a high level of C14ORF166 protein expression was associated with significantly poorer 5-year overall survival (OS, *P* < 0.001; Fig. 4a) and 5-year disease-free survival (DFS, *P* < 0.001; Fig. 4b) in patients with cervical cancer. We observed that the cumulative 5-year overall survival rate was 87 % (95 % CI, 86.9~87.1 %) in the low C14ORF166 group; whereas, it was only 65 % (95 % CI, 67.6~68.0 %) in the high C14ORF166 group.

In subgroup analysis, high-level expression of C14ORF166 was associated with significantly poorer OS in patients without pelvic lymph node metastasis (*P* = 0.041; Fig. 4c) and patients with stages I and II disease (*P* = 0.027; Fig. 4d) but not in patients with stages III and IV disease (*P* = 0.414; data not shown); the lack of a statistically significant association may be due to the limited number of patients in each subset.

Moreover, univariate Cox regression and multivariate survival analysis indicated that C14ORF166 expression (*P* = 0.036), FIGO stage (*P* = 0.009), recurrence (*P* = 0.003), and pelvic lymph node metastasis (*P* = 0.013) were independent prognostic factors for poor overall survival (Table 4). Therefore, these findings indicate that C14ORF166 may represent a valuable prognostic marker in cervical cancer.

**Table 4:** Univariate and multivariate analyses of prognostic factors in cervical cancer using a Cox regression model

	Univariate analysis			Multivariate analysis		
	No. of patients	*P*	Regression coefficient (SE)	*P*	Relative risk	95 % confidence interval
Pelvic lymph node metastasis						
Absent	115	<0.001	1.937 (0.453)	0.013	3.693	1.311–10.403
Present	33					
FIGO stage						
I	51					
II	80	<0.001	1.155 (0.309)	0.009	2.475	1.251–4.898
III	15					
IV	2					
C14ORF166						
Low expression	89	<0.001	2.104 (0.503)	0.036	4.017	1.095–14.731
High expression	59					
Recurrence		0.004	1.396 (0.485)	0.003	4.419	1.659–11.768
No	133					
Yes	15					

## Discussion

In this study, we provide the first evidence that increased expression of C14ORF166 is a common feature and may represent a potential prognostic marker in cervical cancer. Interestingly, C14ORF166 protein expression was also significantly associated with pelvic lymph node metastasis, clinical stage, vital status, tumor size, and serum squamous cell carcinoma antigen level.

In the present study, overexpression of C14ORF166 was not only observed in HPV-positive cervical cancer cell lines, such as HeLa, SiHa, and CasKi, but also in the HPV-negative cervical cancer cell line C33A. Moreover, immunohistochemical analysis revealed that C14ORF166 was overexpressed in the majority of human cervical cancer tissue specimens tested, in agreement with previous studies in brain cancer [10] and pancreatic cancer [11, 12]. However, the molecular mechanisms responsible for overexpression of C14ORF166 in cancer remain largely unknown.

A number of potential biomarkers, including tissue polypeptide antigen (TPA) and tissue polypeptide-specific antigen (TPS) have been suggested to have prognostic value in cervical cancer; however, the data concerning these antigens is conflicting and further studies are required [16–18]. In this cohort, we showed that aberrant expression of C14ORF166 protein was associated with significantly poorer 5-year OS and DFS. Multivariate Cox regression analysis revealed that a high level of C14ORF166 was an independent prognostic marker for cervical cancer. Taken together, our study indicated that C14ORF166 could be a novel prognostic biomarker to identify cervical cancer patients with poor clinical outcome.

In this study, high expression of C14ORF166 protein correlated significantly with advanced tumor stage, larger tumor size, death, lymph node metastasis, and poorer survival, suggesting that C14ORF166 may not only play a role in promoting the growth of the primary tumor but may also be associated with the development of lymph node metastasis in cervical cancer. Associations between C14ORF166 protein expression and the clinical features of other types of cancer have been reported. Guo et al. [11] and Cur et al. [12] showed that expression of C14ORF166 was significantly associated with an aggressive phenotype in pancreatic carcinoma, and Wang et al. [10] reported that expression of C14ORF166 was associated with poor prognostic factors in brain cancer. This evidence indicates that C14ORF166 may play a role in the pathogenesis of various types of cancer.

Recently, Chen et al. [13] investigated the JAK2/STAT3 pathway in esophageal cancer using immunoprecipitation assays and demonstrated C14ORF166 was a binding partner of JAK2. JAK2, a member of the Janus (JAK) family of nonreceptor protein tyrosine kinases, functions as a prototypical kinase to mediate the phosphorylation of signal transducer and activator of transcription 3 (STAT3), thus regulating the JAK2/STAT3 signaling pathway [19, 20]. Activation of the JAK2/STAT3 pathway is associated with inflammation, and inflammation subsequent to viral infection is one factor that initiates and promotes the development of cancer [21–25]. Hyperactivation of inflammatory pathways plays an important role in cervical cancer tumorgenesis, and promotes progression from low-grade lesions to invasive cervical cancer [26]. Additionally, Sobti et al. [27] indicated a potentially interactive effect between HPV16/18 and activation of the JAK2/STAT3 pathway in cervical carcinogenesis. Furthermore, it was reported that the JAK2/STAT3 pathway regulates cervical cancer progression [14, 15]. These studies indicate that overexpression of C14ORF166 may affect the development and progression of cervical cancer by modulating the JAK2/STAT3 signaling pathways; further characterization of these processes may provide new insight and novel targets for the treatment of cervical cancer. The molecular mechanism that links C14ORF166 to tumor progression in cervical cancer remains to be elucidated, and further studies are warranted.

It is widely recognized that various treatment strategies lead to different clinical outcomes in patients with cervical cancer. Currently, assessment of lymph node metastasis provides important information for determining the appropriate treatment approach, such as surgical resection and chemoradiation [28, 29]. Moreover, lymph node metastasis is an important cause of cervical cancer-related mortality [30]. Noordhuis et al. [31] found that patients with early-stage cervical cancer who did not have lymph node metastasis had a 5-year overall survival rate of 90 % compared to only 65 % for patients with lymph node metastasis. However, there are no accurate and efficient techniques for diagnosing pelvic lymph node metastasis to help gynecologic oncologists select the appropriate treatment and avoid unnecessary surgical intervention [32]. Interestingly, we observed that C14ORF166 was significantly associated with pelvic lymph node metastasis (*P* < 0.001). In accordance with these results, Guo et al. [11] and Cui et al. [12] previously reported that C14ORF166 was a novel lymph node metastasis-associated protein in pancreatic cancer. Hence, we suggest that C14ORF166 has potential as a novel predictor of pelvic lymph node metastasis; additional multi-center prospective studies are required to validate this hypothesis. Additionally, further study of the role of C14ORF166 in invasion and metastasis may help to clarify the mechanisms that regulate metastasis and identify novel therapeutic targets for cervical cancer.

Squamous cell carcinoma antigen (SCC-Ag) is commonly employed in the clinic for cervical cancer screening and as an important prognostic factor for patient survival [33]. High serum SCC-Ag levels have been associated with pelvic lymph node metastasis, tumor stage, tumor size, recurrence, and survival in cervical cancer [34–36]. However, SCC-Ag is neither cervix-specific nor malignancy-specific; the serum levels of SCC-Ag are significantly elevated in patients with lung cancer, esophageal squamous cell carcinoma, and head and neck squamous cell carcinoma [37–39]. Torre et al. [40] also indicated that serum SCC-Ag was elevated in patients with benign conditions including tuberculosis, eczema, and pemphigus. Kim et al. [41] demonstrated that increased serum SCC-Ag (>2.0 ng/mL) may be predictive for lymph node metastases in early-stage cervical carcinoma, and Takeda et al. [42] reported that serum SCC-Ag levels over 1.5 ng/ml were significantly associated with lymph node metastasis. However, the cutoff levels for SCC-Ag varied from study to study resulting in vastly different sensitivity and specificity values for this marker [34, 43, 44], and a normal pretreatment SCC-Ag level does exclude the presence of lymph node metastases in cervical cancer [45]. Therefore, the predictive value of serum SCC-Ag for pelvic lymph node metastases in cervical carcinoma is unsatisfactory [17]. Herein, we found that C14ORF166 was strongly associated with and may therefore represent a valuable biomarker of pelvic lymph node metastasis. Thus, C14ORF166 may be useful for evaluating prognosis and guiding follow-up therapy in cervical cancer. Additionally, C14ORF166 can be detected in the serum of patients with pancreatic cancer [9]; however, we did not explore the expression of C14ORF166 in the serum of patients with cervical cancer. Therefore, further investigation is required to confirm whether C14ORF166 has potential as predictive biomarker for identifying patients with pelvic lymph node metastasis.

## Conclusion

In our study, we report that the expression of C14ORF166 is upregulated in cervical cancer cells and human surgical specimens. Additionally, C14ORF166 was significantly associated with pelvic lymph node metastasis, clinical stage, tumor size, vital status, and the serum squamous cell carcinoma antigen level. Patients with highly expressed C14ORF166 showed a tendency to receive postoperative chemotherapy and postoperative radiation. Multivariate analysis revealed that C14ORF166 was an independent prognostic factor for cervical cancer. Taken together, these results suggest that C14ORF166 may play an important role in the development and progression of human cervical carcinoma. C14ORF166 may represent a potential biomarker for lymph node metastasis in cervical cancer, which may help to identify patients at high risk and establish a rationale for selecting appropriate therapeutic strategies.
